# A conceptual classification scheme of invasion science

**DOI:** 10.1093/biosci/biae093

**Published:** 2024-10-26

**Authors:** Camille L Musseau, Maud Bernard-Verdier, Tina Heger, Leonidas H Skopeteas, David Strasiewsky, Daniel Mietchen, Jonathan M Jeschke

**Affiliations:** Department of Evolutionary and Integrative Ecology, Leibniz Institute of Freshwater Ecology and Inland Fisheries (IGB); Department of Biology, Chemistry, Pharmacy, Institute of Biology, at Freie Universität Berlin (FUB); Berlin-Brandenburg Institute of Advanced Biodiversity Research (BBIB), Berlin, Germany; Department of Evolutionary and Integrative Ecology, Leibniz Institute of Freshwater Ecology and Inland Fisheries (IGB); Department of Biology, Chemistry, Pharmacy, Institute of Biology, at Freie Universität Berlin (FUB); Berlin-Brandenburg Institute of Advanced Biodiversity Research (BBIB), Berlin, Germany; Department of Evolutionary and Integrative Ecology, Leibniz Institute of Freshwater Ecology and Inland Fisheries (IGB); Department of Biology, Chemistry, Pharmacy, Institute of Biology, at Freie Universität Berlin (FUB); Berlin-Brandenburg Institute of Advanced Biodiversity Research (BBIB), Berlin, Germany; Technical University of Munich, TUM School of Life Sciences, Freising, Germany; Department of Evolutionary and Integrative Ecology, Leibniz Institute of Freshwater Ecology and Inland Fisheries (IGB); Department of Biology, Chemistry, Pharmacy, Institute of Biology, at Freie Universität Berlin (FUB); Berlin-Brandenburg Institute of Advanced Biodiversity Research (BBIB), Berlin, Germany; Department of Evolutionary and Integrative Ecology, Leibniz Institute of Freshwater Ecology and Inland Fisheries (IGB); Department of Biology, Chemistry, Pharmacy, Institute of Biology, at Freie Universität Berlin (FUB); Berlin-Brandenburg Institute of Advanced Biodiversity Research (BBIB), Berlin, Germany; Department of Evolutionary and Integrative Ecology, Leibniz Institute of Freshwater Ecology and Inland Fisheries (IGB); Department of Biology, Chemistry, Pharmacy, Institute of Biology, at Freie Universität Berlin (FUB); Berlin-Brandenburg Institute of Advanced Biodiversity Research (BBIB), Berlin, Germany; Ronin Institute for Independent Scholarship, Montclair, New Jersey, United States; Institute for Globally Distributed Open Research and Education, Jena, Germany; Department of Evolutionary and Integrative Ecology, Leibniz Institute of Freshwater Ecology and Inland Fisheries (IGB); Department of Biology, Chemistry, Pharmacy, Institute of Biology, at Freie Universität Berlin (FUB); Berlin-Brandenburg Institute of Advanced Biodiversity Research (BBIB), Berlin, Germany

**Keywords:** biological invasions, hierarchy-of-hypotheses (HoH) approach, knowledge mapping, meta-invasion science, research themes

## Abstract

In the era of big data and global biodiversity decline, there is a pressing need to transform data and information into findable and actionable knowledge. We propose a conceptual classification scheme for invasion science that goes beyond hypothesis networks and allows to organize publications and data sets, guide research directions, and identify knowledge gaps. Combining expert knowledge with literature analysis, we identified five major research themes in this field: introduction pathways, invasion success and invasibility, impacts of invasion, managing biological invasions, and meta-invasion science. We divided these themes into 10 broader research questions and linked them to 39 major hypotheses forming the theoretical foundation of invasion science. As artificial intelligence advances, such classification schemes will become important references for organizing scientific information. Our approach can be extended to other research fields, fostering cross-disciplinary connections to leverage the scientific knowledge needed to address Anthropocene challenges.

In the 1980s, John Naisbitt argued that “we are drowning in information but starved for knowledge” (Naisbitt 1982, p. 24). Four decades later, this statement holds even greater relevance. Transforming vast amounts of data and information into actionable knowledge is a pressing challenge (Jeschke et al. 2019), particularly in scientific domains addressing the current decline in biodiversity (Balbi et al. 2022). The substantial increase in information makes it difficult for researchers and practitioners to acquire and maintain an overview of the field, leading to challenges such as accessing existing evidence and ensuring effective knowledge transfer (Jeschke et al. 2021). Artificial intelligence (AI) has become a critical tool for structuring and extracting meaningful knowledge from vast data. With its ability to analyze complex information, AI unveils hidden patterns, trends, and relationships. Harnessing the potential of AI for science is therefore advisable, but it is essential that human experts stay in full control of the scientific conclusions drawn from any output. Current and future research should strive for the most useful and reliable combinations of human expertise with AI-based analyses. When it comes to providing an overview on a research field, we suggest that a good way forward is combining robust conceptual frameworks developed from human expertise and contextual understanding with AI-based tools for information retrieval (Suominen and Toivanen 2016). This way, it will be possible to convert data into actionable knowledge, ensuring that the produced insights are relevant, reliable, and applicable to real-world challenges.

Biological invasions trigger biodiversity loss worldwide (Roy et al. 2024). The number of non-native species, commonly defined as species being introduced through human intervention to one or more regions where they are not native, has significantly increased, with no signs of slowing down (Seebens et al. 2017). This development has made it clear that effective management and policy strategies are urgently needed to prevent future introductions and mitigate the impacts of non-native species (Pyšek et al. 2020, Robertson et al. 2020). Invasion science is a relatively young discipline that studies non-native and invasive species. It is a multidisciplinary field that draws on various areas of study, such as ecology and evolutionary biology (Sax et al. 2007), conservation biology (Blackburn et al. 2019), and socioeconomics (Diagne et al. 2021, Heger et al. 2021). This field has become very productive in recent decades, with an exponential increase in published data and information since 2000 (Campbell and Simberloff 2022). Structuring this information with a robust classification scheme enhances access to knowledge, improving research and management efficiency—a crucial task to better protect biodiversity and ecosystem services facing biological invasions (Enders et al. 2020, Jeschke et al. 2021, Stevenson et al. 2023).

As a discipline with a strong theoretical foundation structured around a series of major hypotheses, invasion science can sometimes be difficult to navigate without a clear overview of past and current theory (Catford et al. 2009, Enders et al. 2018, 2019). Conceptual maps of the discipline have been recently developed along the timeline of invasion, building on hypotheses associated with stages of invasion (Daly et al. 2023). Previous works have explored whether these hypotheses are supported by empirical evidence and aimed to better understand their interrelationships (Jeschke and Heger 2018). Enders and colleagues (2020) created a conceptual map of the discipline with a network of 39 hypotheses on the basis of a consensus approach by experts’ knowledge; this map visualizes the similarities and dissimilarities of hypotheses and helps to identify main theoretical clusters structuring the field (https://hi-knowledge.org/invasion-biology-large). However, as in other research fields, not all research done in invasion science addresses established hypotheses. To be able to structure and organize data and information in invasion science and other research fields, we therefore need to go beyond hypothesis networks.

A promising way forward is to create hierarchical conceptual schemes that capture the major themes, research questions, and hypotheses of a research field. Such schemes will allow to structure and organize all the information available within research fields. They should be made openly available—for example, through a dynamic database or information management system and set up in a way that they can be further expanded and refined as new information becomes available, ideally by the whole community. Publications, data sets and other information should be linked to the corresponding themes and research questions plus the hypotheses where applicable. This would help users to easily find and access relevant information, to identify research gaps, and to develop future research directions.

Using invasion science as a case example, we developed such a new classification scheme. This classification scheme goes beyond existing overviews and networks because it explicitly includes research in invasion science that is not only based on major hypotheses. In the present article, we describe the methodological steps for its development, coupling experts’ knowledge and a literature review. Although the scheme is focused on invasion science, the methodology can also be applied to other research fields, which would create the promising opportunity to develop a multidisciplinary scheme as a next step.

## Identifying themes and research questions linked to hypotheses

**Figure 1.**
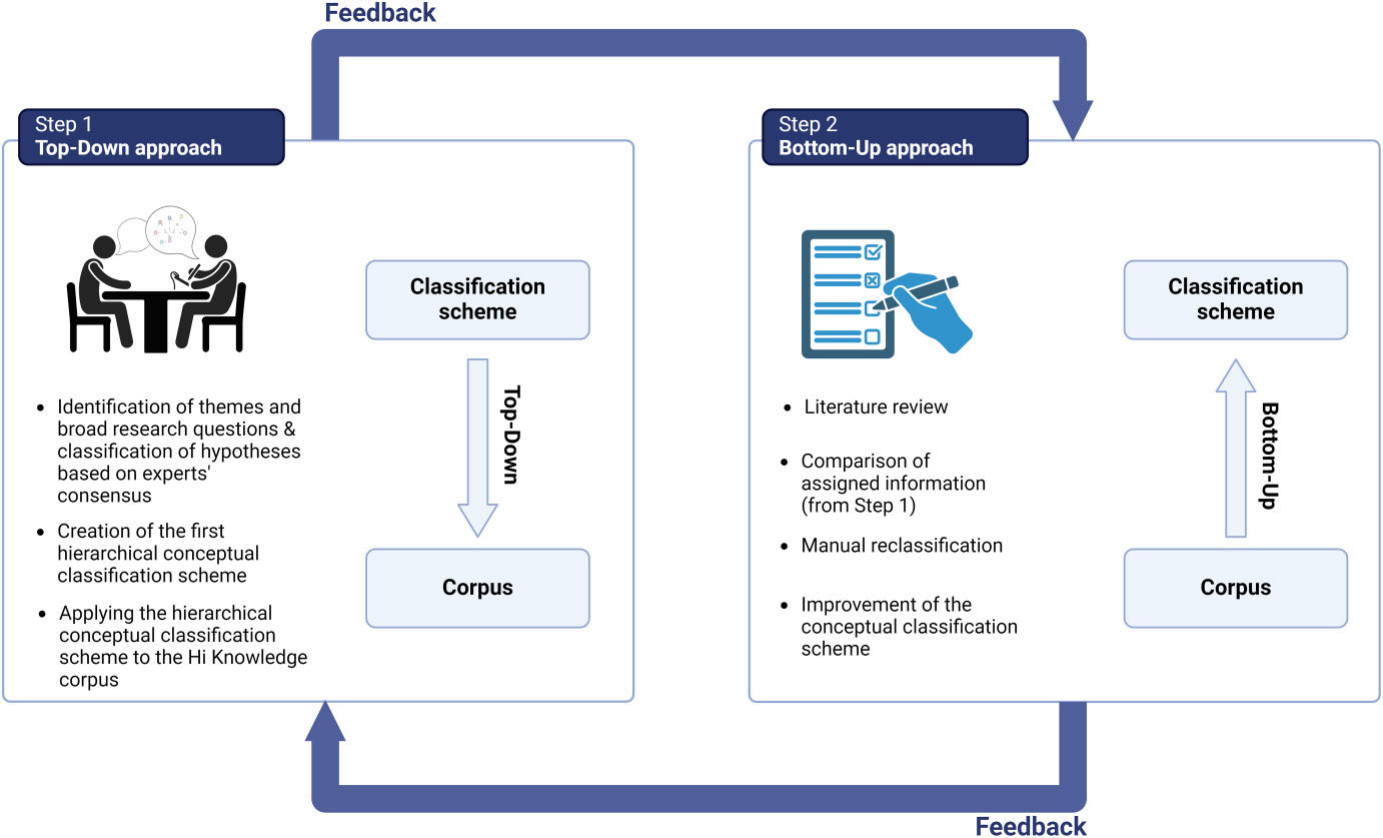
Workflow describing the methodology used to develop a hierarchical conceptual classification scheme for invasion science, consisting of a top-down and a bottom-up approach.

To develop a conceptual classification scheme encompassing all overarching themes and research questions in the field of invasion science, we combined a top-down with a bottom-up classification approach (figure 1). We focused on 39 major hypotheses forming the theoretical base of the discipline (supplemental table S1). This list is not exhaustive or final but serves as a starting point; it includes hypotheses selected through a prior consensus among experts (Enders et al. 2020).

The term *invasive species* has different definitions, and this terminology is the subject of an ongoing debate within the community. While the Convention on Biological Diversity (2010) defined *invasive species* as non-native species spreading beyond their natural ranges and threatening biological diversity, other definitions do not consider the impacts of those species to define their invasiveness (i.e., Ricciardi and Cohen 2007). In the present article, we avoided the term *invasive* in our themes and questions and, rather, distinguished invasion success, as *establishment and spread*, from *impact*. We classified hypotheses on the basis of their original formulation and context, accounting for diverse and ambiguous terminology. The term *invasive* is used in only three hypothesis definitions (colonization pressure, sampling, plasticity) and always with the meaning of “spreading,” without obvious implications of impact. We interpreted the presence of impact from the words *ecological impact, harm, affects them negatively*, and *consecutive steps of invasion* (table S1). By acknowledging and incorporating the various viewpoints, we aimed to ensure that our scheme is applicable for the whole field, accommodating the range of perspectives on invasiveness.

### Top-down approach

To identify the most relevant research questions in invasion science, we relied on the expert knowledge of a subset of the authors (CLM, JJ, MBV, and TH). In several sessions, we first formulated a list of all possible overarching research questions in invasion science, which we then classified hierarchically within major themes, and finally used a consensus approach to connect an existing list of 39 hypotheses (table S1) to both the research questions and themes (figure 1). Each expert filled in a table indicating whether a given hypothesis was connected or not to any of the research questions and their associated themes. The guideline was to restrict ourselves to the historical definition, focusing on connecting hypotheses to the question(s) and theme(s) in which they were historically and theoretically developed. The individual classification schemes were then collected and compared to identify consistencies and inconsistencies among the experts (supplemental table S2). In a second round, the experts reassessed their classification and focused on the hypothesis-research question pairs that had received inconsistent assessments. The final classification scheme was then compiled through a discussion among the experts to reach a consensus (table S2). To assess uncertainty among experts’ scores, we conducted an interrater reliability analysis using Krippendorff's *α* (*kripp.alpha* function, irr R package; Gamer et al. 2019). This index ranges from 0 (indicating no reliability) to 1 (indicating perfect reliability) and provides an overview of the main sources of debate among the experts for each hypothesis (table S2).

### Bottom-up approach

After completing the initial top-down approach, we applied a bottom-up approach. It consisted of a manual classification of studies according to the developed hierarchical classification scheme (undertaken by CLM, LHS and DS). Our aim was to check whether the list of themes and research questions identified in the top-down approach covers the full range of topics addressed in the literature. We had access to a corpus of approximately 1100 studies previously identified as testing well-known hypotheses in invasion science (Jeschke and Heger 2018). We used the new conceptual scheme to automatically assign each study to research questions and major themes on the basis of the hypothesis they were testing. We focused on a subset of 289 publications addressing 10 invasion hypotheses: the biotic resistance hypothesis (*n* = 30 publications; Elton 1958, Levine and D'Antonio 1999), Darwin's naturalization hypothesis (*n* = 30; Darwin 1859, Daehler 2001), the disturbance hypothesis (*n* = 30; Elton 1958, Hobbs and Huenneke 1992), the enemy release hypothesis (*n* = 30; Keane and Crawley 2002, Heger et al. 2024), the invasional meltdown hypothesis (*n* = 29; Simberloff and Von Holle 1999), the island susceptibility hypothesis (*n* = 13; Jeschke 2008), the limiting similarity hypothesis (*n* = 32; MacArthur and Levins 1967), the plasticity hypothesis (*n* = 32; Richards et al. 2006), the propagule pressure hypothesis (*n* = 33; Lockwood et al. 2005), and the tens rule (*n* = 30; Williamson and Brown 1986; see references and definitions in table S1). For each of these 289 papers, we examined which research questions and themes it addresses and checked for possible additional hypotheses it might also cover (supplemental table S3). We then compared this manual classification with the themes and research questions expected on the basis of the hypothesis tested and the first version of the conceptual scheme created using the top-down approach (step 1).

Different types of inconsistencies between the top-down and bottom-up classifications were possible: major connections between hypotheses and questions or themes, which we had overlooked in the top-down approach; major questions or themes that we had overlooked completely; erroneous hypothesis assignment in the original corpus; and a loose connection between an hypothesis and a research question, revealing a secondary application of the hypothesis outside of its primary context. Inconsistencies were discussed and informed a new round of revisions of the top-down approach (i.e., for the first or second type of inconsistency). Further feedback on preliminary versions of the scheme was provided by other invasion biologists at two international conferences and a workshop in 2023 (Bernard-Verdier et al. 2023).

## Conceptual classification scheme

**Figure 2.**
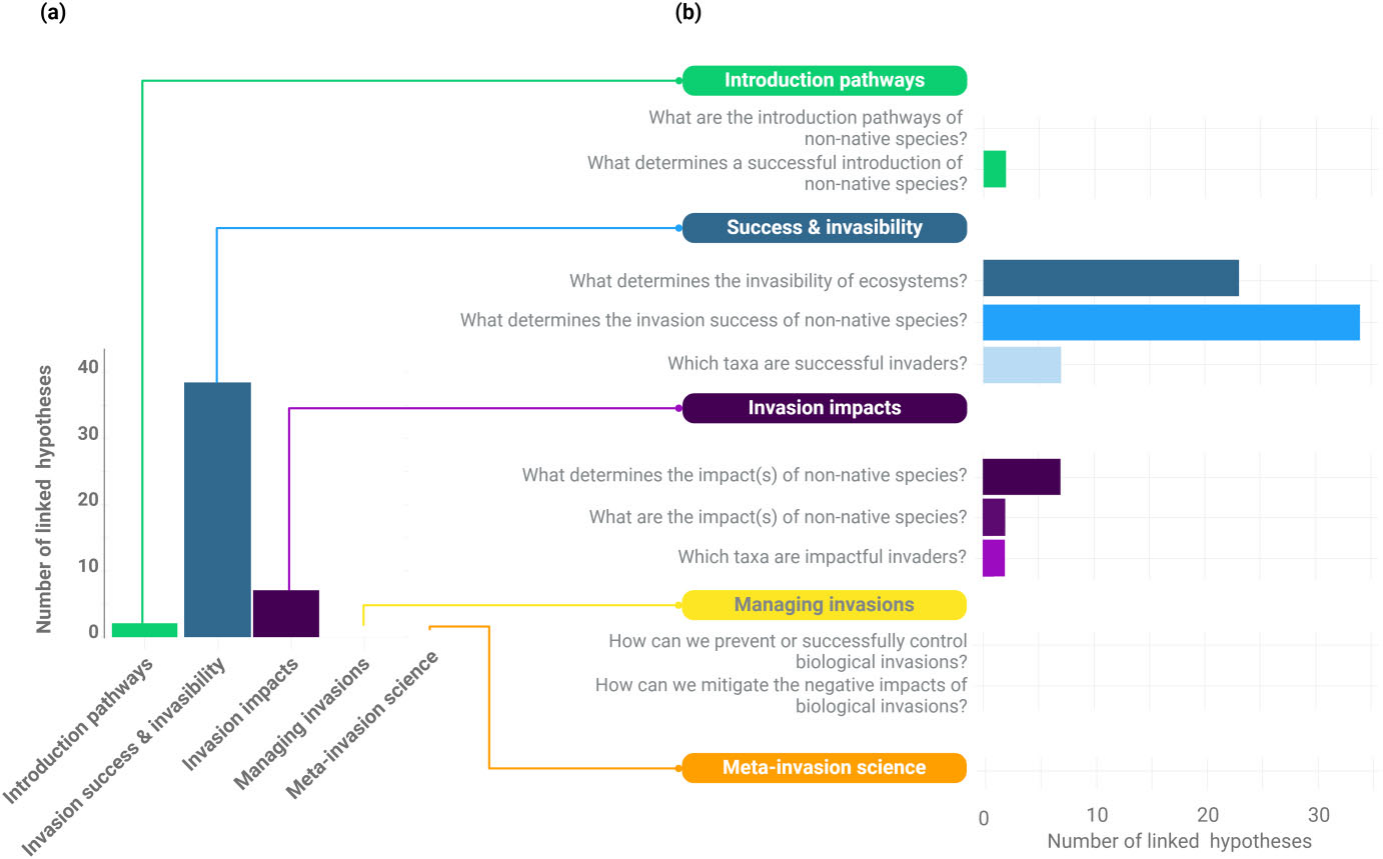
The number of hypotheses linked to (a) each of the five themes and (b) the 10 research questions.

The final conceptual scheme consisted of five major themes, 10 general research questions, and their connections to 39 invasion hypotheses (figure 2). The themes were introduction pathways, invasion success and invasibility, impacts of invasion, managing invasions, and meta-invasion science. The last theme was added after the bottom-up approach revealed a missing major theme. Similarly, although nine of the research questions were formulated a priori in the top-down approach (figure 2), the tenth question (*Which taxa are impactful invaders?*) was added on the basis of the bottom-up approach. We found that all 39 hypotheses addressed the theme of invasion success and invasibility, with some, in addition, addressing invasion impacts (7) or introduction pathways (2), but none directly connected to managing invasions or meta-invasion science (figure 2).

**Figure 3.**
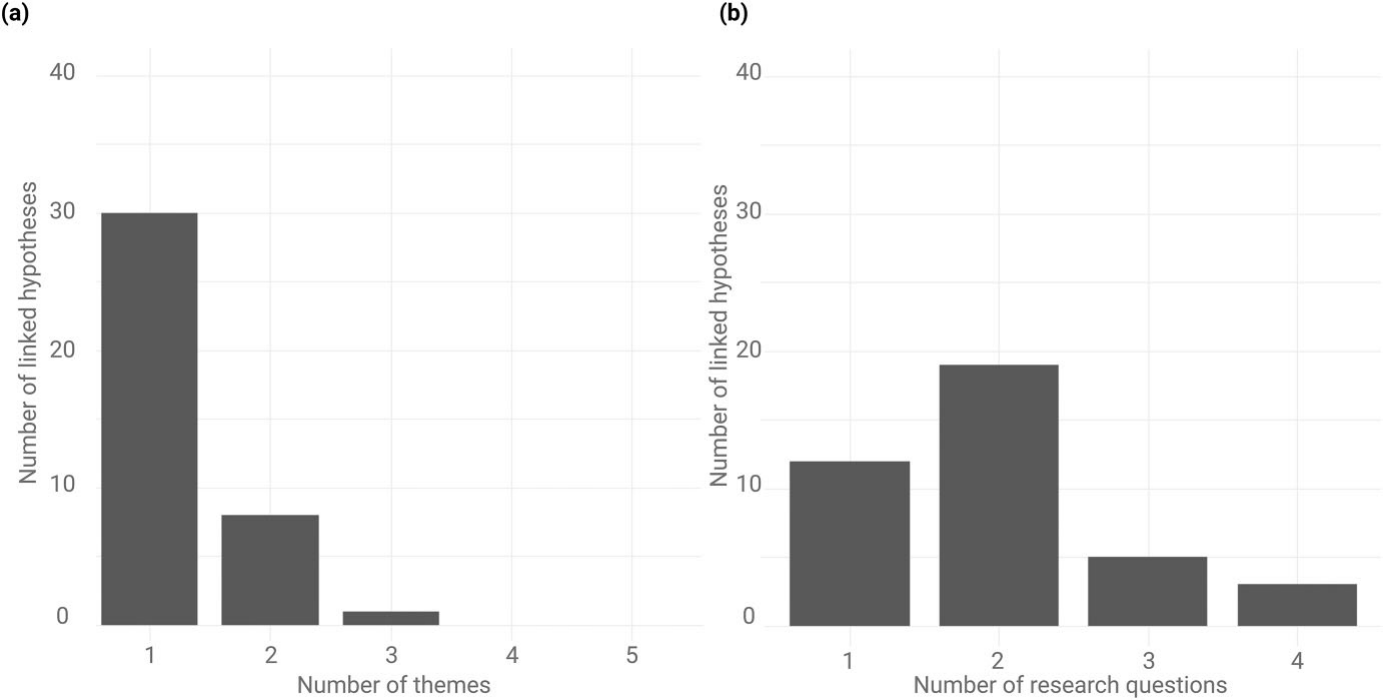
Frequency distributions of individual hypotheses linked to (a) themes and (b) research questions.

Most of the hypotheses (30 of 39) were focused on a single theme, whereas 8 hypotheses were related to two different themes, and one hypothesis (the tens rule) was related to three themes (figure 3a). The hypotheses were connected to at least one and up to four of the research questions (figure 3b). This suggests that although most hypotheses were focused on one specific topic, some of them were broader in their scope, exploring multiple themes and research questions. In the following, we describe the five major themes and the 10 broader research questions. More information on each of the connected hypotheses, including their full names, definitions and key references can be found in table S1.

## Theme 1: Introduction pathways (including pathway conditions)

Introduction pathways encompass the many factors responsible for species’ transport outside of their native range, leading to their introduction in recipient ecosystems (Roy et al. 2024). They are directly related to historical and current international trade and peoples’ migrations that globally displace—intentionally or not—non-native species, having direct and indirect effects on their introductions (Hulme 2021). We identified two main questions structuring this theme.

### What are the introduction pathways of non-native species?

**Figure 4.**
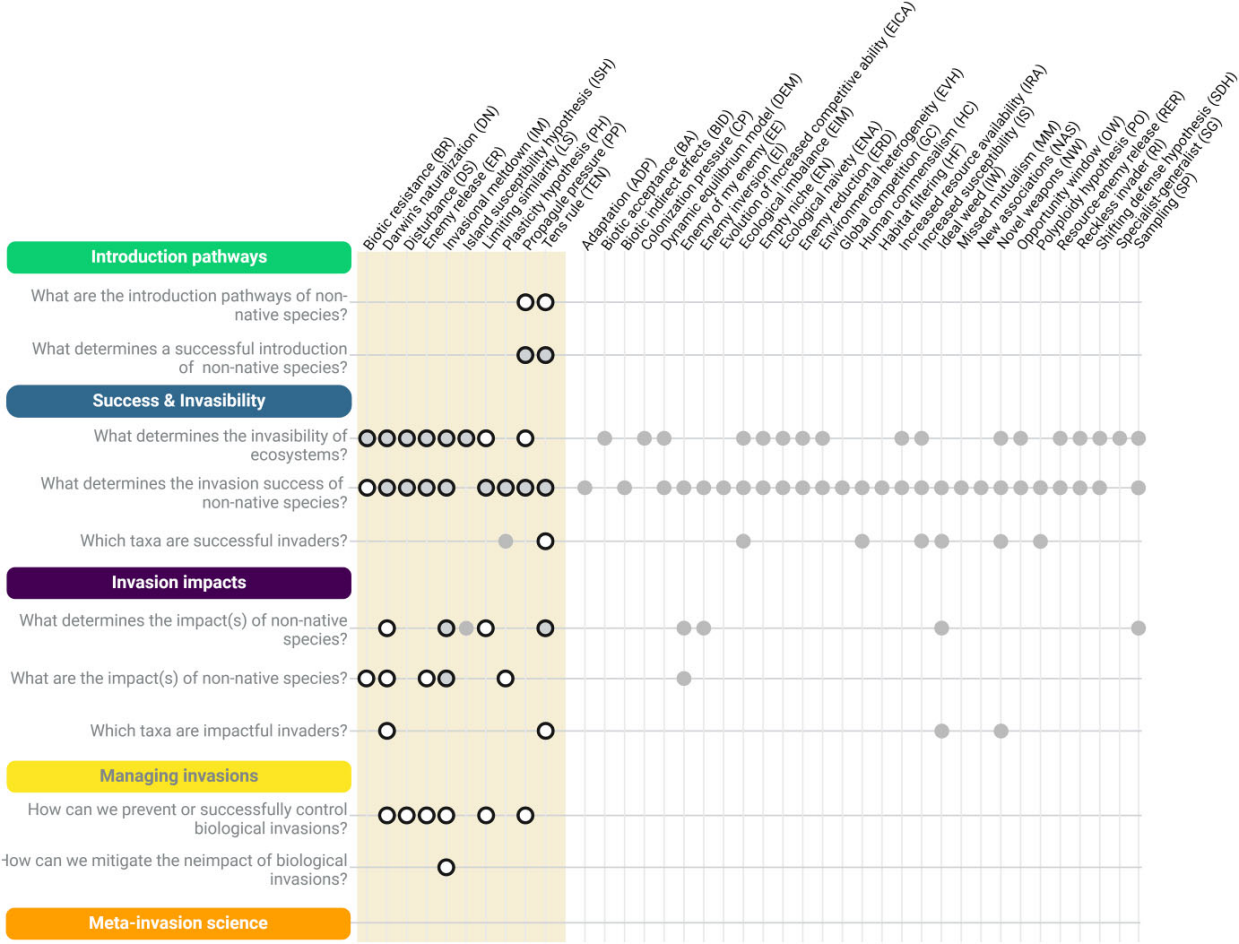
Identified connections between the 39 hypotheses and the 10 research questions. The theoretical, top-down approach was applied for all hypotheses, and the bottom-up approach was also applied for the 10 hypotheses on the left (indicated by the colored background); the full grey circles represent connections identified solely through the top-down approach, the black line open circles represent connections identified only with the bottom-up approach, and the black line grey circles represent connections identified through both approaches. For references and definitions, see supplemental table S1. An interactive version of the conceptual scheme is available here: https://maudbernardverdier.shinyapps.io/Classification-scheme-invasion-science/.

From ballast water to the trade of organisms, introduction pathways across the globe are numerous (Hulme et al. 2008, Saul et al. 2017). Identifying these pathways is crucial for assessing and managing the risks associated to non-native species introductions and to better implement interception policies, such as preborder management and border controls as a preventing way for intercepting the species. None of the 39 hypotheses was linked to this question from a theoretical, top-down perspective (figures 2 and 4). In the bottom-up approach, however, we identified studies addressing the propagule pressure hypothesis and the tens rule that were connected to this research question.

### What determines a successful introduction of non-native species?

Once individuals of a species are transported and introduced beyond the species’ native range, they can potentially survive, which constitutes a successful introduction. Two hypotheses, the propagule pressure hypothesis and the tens rule, were found to directly address this question both in the top-down and bottom-up approaches (figure 4).

## Theme 2: Invasion success (establishment and spread) and invasibility

Invasion success is the ability of a non-native species to establish, persist, and spread in a new environment. Identifying the factors that determine invasion success has been central in invasion science. At the same time, understanding what makes an ecosystem more susceptible to invasions, known as *invasibility*, is intimately connected to invasion success and was grouped under the same theme (Alpert et al. 2000). All 39 hypotheses in this study were linked to this theme, covering three major research questions related to invasion success and invasibility.

### What determines the invasibility of ecosystems?

Determining the factors that make ecosystems vulnerable to biological invasions has long been a principal challenge in ecology (Alpert et al. 2000). The invasibility of an ecosystem can be determined by a variety of factors, both intrinsic and external to that ecosystem, biotic or abiotic (Alpert et al. 2000). We identified 23 hypotheses addressing this major question, with four of them being uniquely related to this question (biotic acceptance biotic resistance, colonization pressure, specialist-generalist).

Multiple hypotheses offer an eco-evolutionary perspective on ecosystem invasibility, highlighting the significance of regional species’ evolutionary legacies in shaping the outcomes of ecosystem invasibility (biotic acceptance, biotic resistance, ecological imbalance, ecological naivety, Darwin's naturalization, island susceptibility, increased susceptibility, novel weapons; Darwin's cluster in Enders et al. 2020). Access to resources by invasive species in recipient ecosystems appears also increased susceptibility as a critical factor determining the potential for ecosystem invasibility (environmental heterogeneity hypothesis, empty niche, dynamic equilibrium model, disturbance hypothesis, increased resource availability, opportunity windows; resource availability cluster in Enders et al. 2020). A set of hypotheses addresses invasibility as a statistical prediction on the basis of the number of species or individuals introduced (colonization pressure, propagule pressure, invasional meltdown, sampling hypothesis; propagule cluster in Enders et al. 2020). Finally, invasibility of ecosystems can be explained by interspecific interactions with resident communities in the invaded range, the dynamics of invasive species being either facilitated or hindered by biotic interactions, contributing to changes in ecosystem invasibility (enemy release, enemy reduction, resource-enemy release, reckless invader, shifting defence, specialist-generalist; biotic interactions cluster in Enders et al. 2020).

### What determines the invasion success of non-native species?

An overwhelming number of hypotheses (34 of 39) were dedicated to understanding the mechanisms that facilitate the successful establishment or spread of non-native species once they were introduced. Eight of the hypotheses were found to be exclusively connected to this facet of invasion success (adaptation, biotic indirect effects, evolution of increased competitive ability, global competition, habitat filtering, limiting similarity, missed mutualisms, new associations).

Invasion success is a multifaceted phenomenon, and numerous hypotheses have been developed to explain the factors and mechanisms contributing to the success of non-native species in new environments, many of which also address invasibility (*What determines the invasibility of ecosystems?*). As above, a key determinant of invasion success is the match between the eco-evolutionary experience of invaders and that of the recipient communities (Darwin's naturalization, empty niche, ecological imbalance, limiting similarity; Darwin's cluster in Enders et al. 2020). Several hypotheses offer insights into how their proximity to humans (human commensalism hypothesis), their specific traits of invaders (adaptation, habitat filtering, increased susceptibility, ideal weed, novel weapons hypothesis; trait cluster in Enders et al. 2020) and their ability to modify those traits (plasticity hypothesis, polyploidy hypothesis) might predict invasion success. Statistical hypotheses suggest that the number of introductions can increase the likelihood of some species or populations finding suitable ecological conditions, which, in turn, affects their potential for successful establishment (global competition, invasional meltdown, propagule pressure, sampling hypothesis, tens rule; propagule cluster in Enders et al. 2020). Hypotheses that focus on environmental heterogeneity and resource accessibility emphasize the role of being able to exploit temporal (disturbance hypothesis, increased resource availability hypothesis) and spatiotemporal fluctuations in resource availability and niche availability (environmental heterogeneity hypothesis, opportunity windows hypothesis) as significant determinants of invasion success. Finally, the ability of an invader to benefit from altered biotic interactions within the recipient communities is often hypothesized to explain invasion success (biotic indirect effects, dynamic equilibrium model, enemy of my enemy, enemy inversion, evolution of increased competititive ability, enemy release, enemy reduction, missed mutualisms, new associations, resource-enemy release, shifting defence; resource availability cluster in Enders et al. 2020), although, in some cases, it may lead to future failure (reckless invader hypothesis).

### Which taxa are successful invaders?

Studies answering the question *Which taxa are successful invaders?* typically aim to identify and list potentially problematic species, and these often include data papers (e.g., Laginhas and Bradley 2022, Martín-Forés et al. 2023). Furthermore, this question is addressed in studies aiming to identify characteristics of species that offer an overall general advantage during the invasion process, regardless of specific contexts or recipient communities. The bottom-up approach revealed the need for this question to be added to our scheme. Publications in our corpus testing the tens rule were often connected to this question, because it provides a statistical prediction (in the percentage of all introduced species) with which empirical lists of introduced and naturalized taxa are often confronted (e.g., Genovesi et al. 2012). Once this research question was added, a second iteration of the top-down approach identified seven hypotheses conceptually linked to this question. These hypotheses addressed either some general attributes characterizing successful invaders in the world (increased susceptibility, ideal weed, novel weapons, plasticity, polyploidy; trait cluster in Enders et al. 2020) or broad historical legacies favoring the selection of invaders (ecological imbalance, human commensalism).

## Theme 3: Impacts of invasion

The impacts of invasion can be defined as changes caused by non-native species in the recipient ecosystems and can be classified as either ecological or socioeconomic (Jeschke et al. 2014). Ecological impacts are the changes following the invasion by non-native species in the biotic and abiotic components of the invaded ecosystem, such as changes in species diversity, species extinctions (Bellard et al. 2016), or ecosystem functions (Pyšek et al. 2020). Socioeconomic changes refer to changes in the human use or management of the ecosystem, such as changes in land use or economic value (Jeschke et al. 2014). The impacts of non-native species on ecosystems can be beneficial, deleterious, or bidirectional (meaning they can have both deleterious and beneficial effects; Simberloff et al. 2013, Jeschke et al. 2014), and they can be measured at different spatial and temporal scales. We identified the following three broad research questions in this theme of invasion impact.

### What determines the impacts of non-native species?

Many research studies aim to determine the impacts of invasions, explain their magnitudes and directions, and identify the mechanisms and characteristics of invaders and recipient ecosystems, which determine the impact. In total, we identified seven hypotheses connected to this question, all of which also address invasion success and consider higher impact as an additional consequence (figure 2). Some hypotheses predict which traits of the invader (ideal weed) or characteristics of the environment (island susceptibility) make negative impacts more likely. Many hypotheses predict the likelihood or size of impact on the basis of mechanisms related to biotic interactions (ecological naivety, enemy inversion, enemy of my enemy, invasional meltdown; biotic interactions cluster in Enders et al. 2020). Finally, one statistical hypothesis predicts a higher likelihood of species having an impact with the number of introductions and successful invasions (tens rule).

### What are the impacts of non-native species?

Invasive species can have diverse impacts on ecosystems at various ecological levels, from individuals to ecosystem functioning, and identifying them is a major concern. Few of the 39 hypotheses in our scheme were distinctly concerned with the different types of impact rather than the likelihood or the size of impact, and initially, our top-down approach identified only two such hypotheses, which both described mechanisms of shifting impacts via cascading biotic interactions (enemy of my enemy, invasional meltdown). However, the bottom-up approach showed that other hypotheses are often used in studies addressing this question (biotic resistance, Darwin's naturalization, enemy release, plasticity). We considered these examples to illustrate a secondary application of these hypotheses to address the question of types of impacts but not the primary context in which the hypotheses were developed.

### Which taxa are impactful invaders?

Studies asking which taxa are impactful invaders typically identify and list known problematic species in a region or globally. Publications listing impactful species, such as data papers or databases, are relevant to this question (e.g., Kolar and Lodge 2001, Ricciardi and Kipp 2008). In contrast to the question *Which taxa are successful invaders?* these studies concern species with known impact, typically applying one of a number of classification schemes that have been developed to assess impacts of non-native species (González-Moreno et al. 2019)—for example, the IUCN EICAT scheme (Environmental Impact Classification for Alien Taxa; Blackburn et al. 2014, IUCN 2020). In addition, some studies try to identify what characteristics generally make an impactful invader. Two hypotheses addressed this question by predicting the general traits, such as life-history characteristics, reproductive strategies, growth rates, and competitive abilities, which are likely to make a problematic invader (ideal weed, novel weapons). The bottom-up approach revealed that—as was the case for the question *Which taxa are successful invaders?—*studies addressing this question often tested the tens rule (figure 4), which provides a statistical prediction, and we therefore added a connection.

## Theme 4: Managing biological invasions

Managing biological invasions involves actions aimed at reducing the presence, abundance, spread, or mitigating the impacts of non-native species. These actions may include preventing a non-native species from entering a new area (prevention and captive management); eliminating it if it has already been introduced but is not yet widely established (eradication); and measures taken if the non-native species is already widely established, either by reducing its spread and abundance (long-term management) or by reducing its per-capita impact (e.g., through restoration measures, impact mitigation, or adaptation; Robertson et al. 2020). None of our 39 hypotheses were primarily formulated for managing biological invasions, but the bottom-up approach revealed examples of application of some theoretical hypotheses to more practical management questions (figure 4). We found two major questions structuring this theme.

### How can we prevent or successfully control biological invasions?

This question addresses studies aiming to control the success of invasive species at the different steps of the invasion process (Robertson et al. 2020). It encompasses prevention strategies, such as the development and application of tools for early-detection monitoring techniques (e.g., Larson et al. 2020), measures to eradicate or reduce invader populations (e.g., Weidlich et al. 2020) or that limit their spread. The bottom-up approach found secondary connections to six hypotheses (Darwin's naturalization, disturbance, enemy release, invasional meltdown, limiting similarity, propagule pressure), which illustrates how studies addressing practical concerns on invasive species control apply and even test these theoretical hypotheses.

### How can we mitigate negative impacts of invasive species?

This second question relates to studies focusing on management measures to reduce the per-capita impact of invasive species. In contrast to the first question, management measures connected to this question do not focus on reducing an invader's abundance or spread by either lethal or nonlethal measures. They do not directly intervene with the invader; therefore, individuals of the invasive species are not being harmed. Examples of such measures include ecological restoration of invaded ecosystems and adaptative strategies or technical solutions that reduce impacts (e.g., raccoon-proof trash bins). In our bottom-up approach, only one hypothesis (the invasional meltdown hypothesis) was found to be associated to this question.

## Theme 5: Meta-invasion science *sensu lato*

Meta-invasion science is a research theme where reflection converges with methodological (Palma et al. 2022), historical (Pyšek and Hulme 2009), philosophical (e.g., the concept of nativeness; Warren 2023), and social dimensions (e.g., social perception of invasive species; Kapitza et al. 2019), collectively enhancing our understanding of the complexities and the ongoing debates within invasion science. Examples of studies connected to this theme include those describing research biases (e.g., Pyšek et al. 2008, Vimercati et al. 2020), citation networks (e.g., Abrahams et al. 2019), critics of the field (e.g., Cassini 2020), and research aiming at synthesizing the discipline (e.g., the present study or Enders et al. 2020). This theme, although not explicitly connected with questions or hypotheses in the present scheme, serves as an essential space for a comprehensive understanding of invasion science.

## A comprehensive organization of invasion science

The suggested conceptual classification scheme of invasion science (figure 4, interactive version: https://maudbernardverdier.shinyapps.io/Classification-scheme-invasion-science) provides an overview of the major themes, research questions, and hypotheses in invasion science, and their interconnectedness. It provides a novel theoretical framework for classifying the publications, data sets, and other information, serving as starting point for a structured overview of the available information. The construction of this scheme involved the development and application of a top-down and a bottom-up approach, leading to the identification of five major themes and 10 overarching research questions linked to 39 hypotheses.

The top-down approach aimed to provide a simplified and expert-curated overview, whereas the bottom-up approach refined this by analyzing a literature corpus (table S3) and assessing the context (i.e., research question and theme) in which the 39 theoretical hypotheses are referred to in the literature. The analysis of a sample set of publications in the bottom-up approach helped us to refine the conceptual scheme previously developed in the top-down approach. With both approaches, assignments of hypotheses to research questions and themes were based on expert opinion. Both approaches are therefore restricted by the fact that hypotheses often have changing and ambiguous meanings (e.g., multiple versions of such major hypotheses typically exist). We addressed this challenge by applying a consensus technique as was described above. However, this highlights the necessity to formalize hypotheses by providing specific verbal definitions, concise descriptions of their components and references to prior work (see Heger et al. 2024, Mietchen et al. 2024).

We found that not all research questions are linked to established hypotheses (figure 2). For instance, questions in the introduction pathway theme (*What are the introduction pathways of non-native species?*), as well as those related to managing biological invasions and meta-invasion science, lacked associated hypotheses. Although research often aims to generate new knowledge, hypotheses in invasion science have been predominantly developed in a fundamental rather than an applied context (e.g., Richards et al. 2006). This may explain the absence of major hypotheses linked to the practical theme of managing invasions. However, hypotheses from fundamental research can be applied as tools for improving invasive species management (Cadotte et al. 2021). The bottom-up approach revealed a broader usage of hypotheses beyond their initial research context (e.g., Dostál and Palečková 2011, Griffiths et al. 2013). Developing a specific classification scheme for management could enhance the organization of knowledge on this theme from a problem-driven perspective.

Recognizing the dynamic nature of scientific research, the conceptualization of themes and questions should continually evolve for both fundamental studies and practical applications. These dynamics in research themes and questions result from evolving research paradigms, emerging methodologies, and the continuously expanding body of literature. An important aspect of this classification is that papers addressing invasion hypotheses often intersect with multiple research questions and themes, depending on the range of invasion processes, mechanisms, and dynamics that a single hypothesis can address, also reflecting the interconnectedness of ecological processes and research interests (Lockwood et al. 2001, Bennett et al. 2014). Moreover, themes themselves may change over time, responding to emerging theories, technological advancements, and shifting ecological priorities. Finally, new hypotheses are constantly being proposed (e.g., Daly et al. 2023). Therefore, we think of the presented scheme as representing a snapshot in the ongoing multidisciplinary research on biological invasions. We made our online scheme interactive and editable, in the hope of inviting the community to propose alternative versions and reach new consensus in the future.

## Implication for an atlas of invasive science

**Figure 5.**
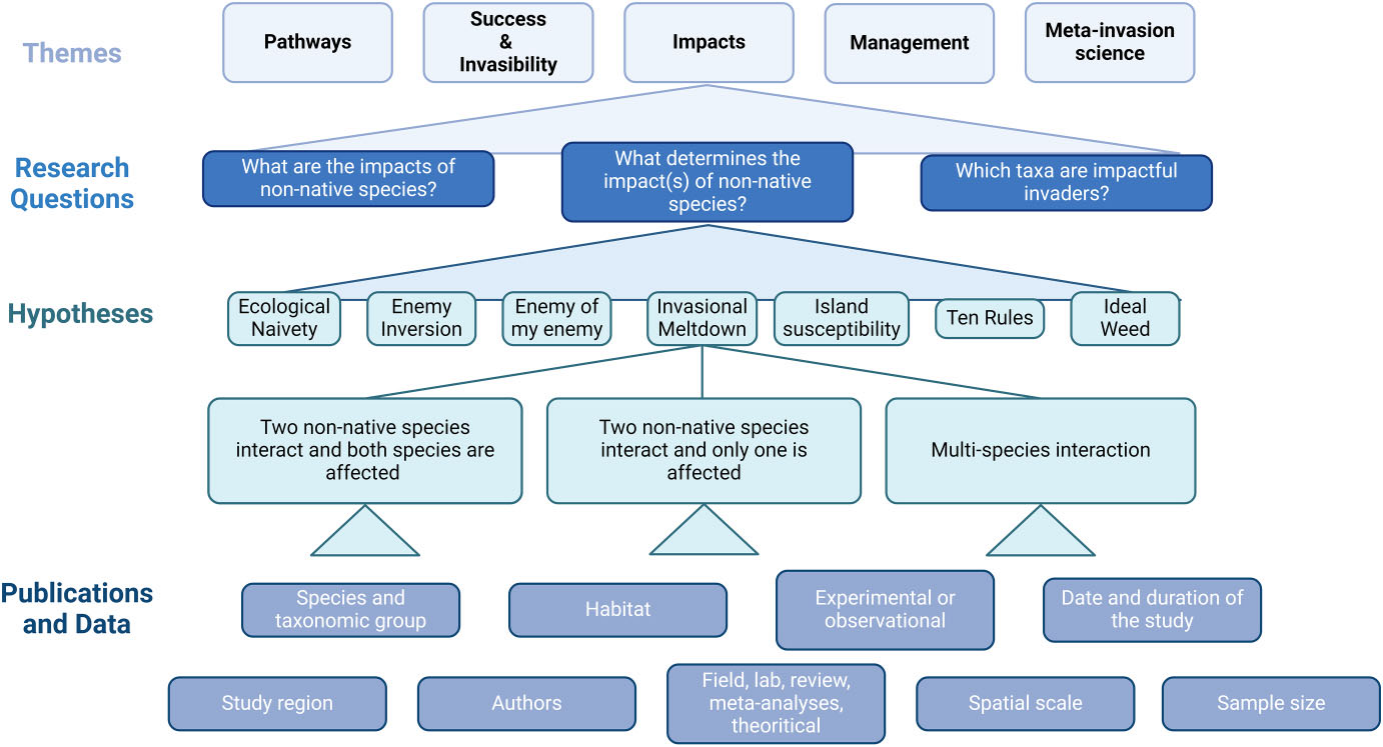
Illustrative example of the conceptual scheme with a focus on the invasional meltdown hypothesis. The hierarchical structure includes (a) all five themes; (b) the three research questions related to the impact theme; (c) hypotheses addressing one of these questions, including invasional meltdown; (d) the three subhypotheses of the invasional meltdown hypothesis; and (e) the relevant publications and data; modified from Jeschke and colleagues (2021).

The suggested conceptual scheme forms a foundation of an open and zoomable atlas of invasion science (figure 5). We envision this atlas to combine various types of information and data visualization to facilitate access to publications and to offer an interactive platform for exploring key themes, questions, and hypotheses in invasion science (Jeschke et al. 2021). A first preliminary version is available at www.hi-knowledge.org, with future improvements planned.

The aim of the envisioned atlas of invasion science is to make invasion science more accessible and navigable for diverse user groups. Users without a background in invasion science, including students and researchers from other fields, will be provided with a broad understanding of the discipline's structure, key topics, publications, and expert contacts. Researchers actively engaged in invasion science will find tools for exploring data, assessing hypotheses robustness, and identifying knowledge gaps in their specific areas of interest. Stakeholders, practitioners, and policymakers involved in invasion management can access pertinent information on research outcomes, species management, ecosystem restoration, and potential collaborators. Educators and communicators, including teachers, professors, and journalists, can leverage the atlas for educational and outreach materials and increase public awareness.

The development of the first conceptual scheme is a preliminary and necessary step toward such an atlas of invasion science. It provides a systematic approach to organize knowledge, serving as a classification system that could be filled with routinely updated scientific information. The implementation of the digital atlas will rely heavily on leveraging AI based tools for information retrieval, semantic modeling, and a mix of manual and automated data curation. Our vision and main principles emphasize open access, limited top-down decisions and a community-curated, noncommercial platform showcasing the plurality and diversity of the discipline. This process will be enabled by an open database facilitated by Wikidata (www.wikidata.org/wiki/Wikidata:Main_Page). The collaborative efforts of AI and open science, as is showcased at www.hi-knowledge.org, provide an interactive representation of scientific information, thereby fostering knowledge transfer and evidence-based decision-making related to biological invasions (Jeschke et al. 2021).

## Expanding the approach to other research fields

We are developing classification schemes for other research fields—for example, restoration ecology (Heger et al. 2022) and urban ecology (Lokatis et al. 2023). Developing conceptual schemes across disciplines can enhance research efficiency and foster interdisciplinary collaborations. Identifying overarching themes and ideas across research fields promise to clarify terminology, avoid redundance, reveal conceptual connections such as shared hypotheses and concepts (Latombe et al. 2021), and identify knowledge gaps and new research opportunities (Jeschke et al. 2021). As AI evolves, robust conceptual classifications based on scientific knowledge become increasingly important to provide expert-curated references and guidance, which can enhance AI tools. This approach can guide researchers through complex information and facilitate the translation of data into actionable insights to address pressing interdisciplinary challenges, such as biodiversity loss, climate change, and public health.

## Supplementary Material

biae093_Supplemental_File The data underlying this article (Table S2 and Table S3) are available in the Dryad Digital Repository, at https://doi.org/10.5061/dryad.9zw3r22q2

## Author Biography

Camille L. Musseau (camille.musseau@igb-berlin.de), Maud Bernard-Verdier, Tina Heger, Leonidas H. Skopeteas, David Strasiewsky, Daniel Mietchen, and Jonathan M. Jeschke are all affiliated with the Department of Evolutionary and Integrative Ecology, in the Leibniz Institute of Freshwater Ecology and Inland Fisheries (IGB), and with the Department of Biology, Chemistry, Pharmacy, in the Institute of Biology, at Freie Universität Berlin (FUB), and with the Berlin-Brandenburg Institute of Advanced Biodiversity Research (BBIB), in Berlin, Germany. Tina Heger is also affiliated with the Technical University of Munich, TUM School of Life Sciences, in Freising, Germany. Daniel Mietchen is also affiliated with the Ronin Institute for Independent Scholarship, in Montclair, New Jersey, in the United States, and with the Institute for Globally Distributed Open Research and Education, in Jena, Germany.
