# Supplementary material for: A conceptual classification scheme of invasion science
Source: Bioscience. 2024 Oct 26;74(12):840–50. doi: 10.1093/biosci/biae093 (PMC11660931; doi:10.1093/biosci/biae093)
Supplement: biae093_Supplemental_File — The data underlying this article (Table S2 and Table S3) are available in the Dryad Digital Repository, at https://doi.org/10.5061/dryad.9zw3r22q2 [file biae093_supplemental_file.docx]

# Table S1- List of 39 common invasion hypotheses and how they were defined for this study (adapted from Enders et al. 2020).

| **Hypothesis** | | **Description** | **Key reference(s)** |
| --- | --- | --- | --- |
| ADP | Adaptation | The invasion success of non-native species depends on the adaptation to the conditions in the exotic range before and/or after the introduction. Non-native species that are related to native species are more successful in this adaptation. | Duncan and Williams 2002 |
| BA | Biotic acceptance aka “the rich get richer” | Ecosystems tend to accommodate the establishment and coexistence of non-native species despite the presence and abundance of native species. | Stohlgren et al. 2006 |
| BID | Biotic indirect effects | Non-native species benefit from different indirect effects triggered by native species. | Callaway et al. 2004 |
| BR | Biotic resistance aka diversity-invasibility hypothesis | An ecosystem with high biodiversity is more resistant against non-native species than an ecosystem with lower biodiversity. | Elton 1958, Levine and D’Antonio 1999 |
| CP | Colonization pressure | Colonization pressure is defined as the number of species introduced to a given location. As colonization pressure increases, the number of established or invasive non-native species in that location is predicted to increase. | Lockwood et al. 2009 |
| DEM | Dynamic equilibrium model | The establishment of a non-native species depends on natural fluctuations of the ecosystem, which influences the competition of local species. | Huston 1979 |
| DN | Darwin’s naturalization | The invasion success of non-native species is higher in areas that are poor in closely related species than in areas that are rich in closely related species. | Darwin 1859, Daehler 2001 |
| DS | Disturbance | The invasion success of non-native species is higher in highly disturbed than in relatively undisturbed ecosystems. | Elton 1958, Hobbs and Huenneke 1992 |
| EIM | Ecological imbalance | Invasion patterns are a function of the evolutionary characteristics of both the recipient region and potential donor regions. Species from regions with highly diverse evolutionary lineages are more likely to become successful invaders in less diverse regions. | Fridley and Sax 2014 |
| ENA | Ecological naivety aka evolutionary naivety | The success and impact of a non-native species on biodiversity is influenced by the eco-evolutionary experience of the invaded community. Thus, the largest impacts are caused by species (e.g. predators, herbivores, pathogens) invading systems where no phylogenetically or functionally similar species exist. | Diamond and Case 1986, Ricciardi and Atkinson 2004 |
| EE | Enemy of my enemy | Introduced enemies of a non-native species are less harmful to the non-native than to the native species. | Colautti et al. 2004 |
| EI | Enemy inversion | Introduced enemies of non-native species are less harmful for them in the exotic than the native range, due to altered biotic and abiotic conditions. | Colautti et al. 2004 |
| EICA | Evolution of increased competitive ability | After having been released from natural enemies, non-native species will allocate more energy in growth and/or reproduction (this re-allocation is due to genetic changes), which makes them more competitive. | Blossey and Notzold 1995 |
| EN | Empty niche | The invasion success of non-native species increases with the availability of empty niches in the exotic range. | MacArthur 1970 |
| ER | Enemy release | The absence of enemies in the exotic range is a cause of invasion success. | Keane and Crawley 2002, Heger et al. 2024 |
| ERD | Enemy reduction | The partial release of enemies in the exotic range is a cause of invasion success. | Colautti et al. 2004 |
| EVH | Environmental heterogeneity | The invasion success of non-native species is high if the exotic range has a highly heterogeneous environment. | Melbourne et al. 2007 |
| GC | Global competition | A large number of different non-native species is more successful than a small number. | Colautti et al. 2006 |
| HC | Human commensalism | Species that live in close proximity to humans are more successful in invading new areas than other species. | Jeschke and Strayer 2006 |
| HF | Habitat filtering | The invasion success of non-native species in the new area is high if they are pre-adapted to this area. | Weiher and Keddy 1995 |
| IM | Invasional meltdown | The presence of non-native species in an ecosystem facilitates invasion by additional species, increasing their likelihood of survival or ecological impact. | Simberloff and Von Holle 1999 |
| IRA | Increased resource availability | The invasion success of non-native species increases with the availability of resources. | Sher and Hyatt 1999 |
| IS | Increased susceptibility | If a non-native species has a lower genetic diversity than the native species, there will be a low probability that the non-native species establishes itself. | Colautti et al. 2004 |
| ISH | Island susceptibility hypothesis | Non-native species are more likely to become established and have major ecological impacts on islands than on continents. | Jeschke 2008 |
| IW | Ideal weed | The invasion success and impacts of a non-native species depends on its specific traits (e.g. life-history traits). | Baker 1965, Rejmanek and Richardson 1996 |
| LS | Limiting similarity | The invasion success of non-native species is high if they strongly differ from native species, and low if they are similar to native species. | MacArthur and Levins 1967 |
| MM | Missed mutualisms | In their exotic range, non-native species suffer from missing mutualists. | Mitchell et al. 2006 |
| NAS | New associations | New relationships between non-native and native species can positively or negatively influence the establishment of the non-native species. | Colautti et al. 2006 |
| NW | Novel weapons | In the exotic range, non-native species can have a competitive advantage against native species because they possess a novel weapon, i.e. a trait that is new to the resident community of native species and therefore affects them negatively. | Callaway and Ridenour 2004 |
| OW | Opportunity windows | The invasion success of non-native species increases with the availability of empty niches in the exotic range, and the availability of these niches fluctuates spatio-temporally. | Johnstone 1986 |
| PH | Plasticity hypothesis | Invasive species are more phenotypically plastic than non-invasive or native ones. | Richards et al. 2006 |
| PO | Polyploidy hypothesis | Polyploid organisms, particularly plants, are predicted to have an increased invasion success, since polyploidy can lead to higher fitness during the establishment phase and/or increased potential for subsequent adaptation. | Te Beest et al. 2012 |
| PP | Propagule pressure | A high propagule pressure (a composite measure consisting of the number of individuals introduced per introduction event and the frequency of introduction events) is a cause of invasion success. | Lockwood et al. 2005 |
| RER | Resource-enemy release | The non-native species is released from its natural enemies and can spend more energy in its reproduction, and invasion success increases with the availability of resources. | Blumenthal 2006 |
| RI | Reckless invader aka “boom-bust” | A population of a non-native species that is highly successful shortly after its introduction can decline or disappear over time due to different reasons (such as competition with other introduced species or adaptation by native species). | Simberloff and Gibbons 2004 |
| SDH | Shifting defence hypothesis | After having been released from natural specialist enemies, non-native species will allocate more energy in cheap (energy-inexpensive) defenses against generalist enemies and less energy in expensive defenses against specialist enemies (this re-allocation is due to genetic changes); the energy gained in this way will be invested in growth and/or reproduction, which makes the non-native species more competitive. | Doorduin and Vrieling 2011 |
| SG | Specialist-generalist | Non-native species are more successful in a new region if the local predators are specialists and local mutualists are generalists. | Callaway et al. 2004 |
| SP | Sampling | A large number of different non-native species is more likely to become invasive than a small number due to interspecific competition. Also, the species identity of the locals is more important than the richness in terms of the invasion of an area. | Crawley et al. 1999 |
| TEN | Tens rule | Approximately 10% of species successfully take consecutive steps of the invasion process. | Williamson and Brown 1986 |

**References**

Baker HG. 1965. Characteristics and modes of origin of weeds. Pages 147–168 in. The genetics of colonizing species; proceedings. Academic Press.

Blossey B, Notzold R. 1995. Evolution of Increased Competitive Ability in Invasive Nonindigenous Plants: A Hypothesis. The Journal of Ecology 83: 887.

Blumenthal DM. 2006. Interactions between resource availability and enemy release in plant invasion. Ecology Letters 9: 887–895.

Callaway RM, Ridenour WM. 2004. Novel weapons: invasive success and the evolution of increased competitive ability. Frontiers in Ecology and the Environment 2: 436–443.

Callaway RM, Thelen GC, Rodriguez A, Holben WE. 2004. Soil biota and exotic plant invasion. Nature 427: 731–733.

Colautti RI, Grigorovich IA, MacIsaac HJ. 2006. Propagule Pressure: A Null Model for Biological Invasions. Biological Invasions 8: 1023–1037.

Colautti RI, Ricciardi A, Grigorovich IA, MacIsaac HJ. 2004. Is invasion success explained by the enemy release hypothesis? Ecology Letters 7: 721–733.

Crawley, Brown, Heard, Edwards. 1999. Invasion‐resistance in experimental grassland communities: species richness or species identity? Ecology Letters 2: 140–148.

Daehler CC. 2001. Darwin’s Naturalization Hypothesis Revisited. The American Naturalist 158: 324–330.

Darwin C. 1859. On the origin of species by means of natural selection, or, The preservation of favoured races in the struggle for life. J. Murray.

Diamond J, Case TJ. 1986. Overview: Introductions, extinctions, exterminations, and invasions. Community ecology. Harper and Row.

Doorduin LJ, Vrieling K. 2011. A review of the phytochemical support for the shifting defence hypothesis. Phytochemistry Reviews 10: 99–106.

Duncan RP, Williams PA. 2002. Darwin’s naturalization hypothesis challenged. Nature 417: 608–609.

Elton CS. 1958. The Ecology of Invasions by Animals and Plants. Springer International Publishing.

Enders M, Havemann F, Ruland F, Bernard‐Verdier M, Catford JA, Gómez‐Aparicio L, Haider S, Heger T, Kueffer C, Kühn I, Meyerson LA, Musseau C, Novoa A, Ricciardi A, Sagouis A, Schittko C, Strayer DL, Vilà M, Essl F, Hulme PE, Van Kleunen M, Kumschick S, Lockwood JL, Mabey AL, McGeoch MA, Palma E, Pyšek P, Saul W, Yannelli FA, Jeschke JM. 2020. A conceptual map of invasion biology: Integrating hypotheses into a consensus network. Global Ecology and Biogeography 29: 978–991.

Fridley JD, Sax DF. 2014. The imbalance of nature: revisiting a D arwinian framework for invasion biology. Global Ecology and Biogeography 23: 1157–1166.

Heger T, Jeschke JM, Bernard-Verdier M, Musseau CL, Mietchen D. 2024. Hypothesis Description: Enemy Release Hypothesis. Research Ideas and Outcomes 10: e107393.

Hobbs RJ, Huenneke LF. 1992. Disturbance, Diversity, and Invasion: Implications for Conservation. Conservation Biology 6: 324–337.

Huston M. 1979. A General Hypothesis of Species Diversity. The American Naturalist 113: 81–101.

Jeschke JM. 2008. Across islands and continents, mammals are more successful invaders than birds. Diversity and Distributions 14: 913–916.

Jeschke JM, Strayer DL. 2006. Determinants of vertebrate invasion success in Europe and North America. Global Change Biology 12: 1608–1619.

Johnstone IM. 1986. Plant invasion windows: a time-based classification of invasion potential. Biological Reviews 61: 369–394.

Keane R, Crawley MJ. 2002. Exotic plant invasions and the enemy release hypothesis. Trends in Ecology & Evolution 17: 164–170.

Levine JM, D’Antonio CM. 1999. Elton Revisited: A Review of Evidence Linking Diversity and Invasibility. Oikos 87: 15.

Lockwood JL, Cassey P, Blackburn T. 2005. The role of propagule pressure in explaining species invasions. Trends in Ecology & Evolution 20: 223–228.

Lockwood JL, Cassey P, Blackburn TM. 2009. The more you introduce the more you get: the role of colonization pressure and propagule pressure in invasion ecology. Diversity and Distributions 15: 904–910.

MacArthur R. 1970. Species packing and competitive equilibrium for many species. Theoretical Population Biology 1: 1–11.

MacArthur R, Levins R. 1967. The Limiting Similarity, Convergence, and Divergence of Coexisting Species. The American Naturalist 101: 377–385.

Melbourne BA, Cornell HV, Davies KF, Dugaw CJ, Elmendorf S, Freestone AL, Hall RJ, Harrison S, Hastings A, Holland M, Holyoak M, Lambrinos J, Moore K, Yokomizo H. 2007. Invasion in a heterogeneous world: resistance, coexistence or hostile takeover? Ecology Letters 10: 77–94.

Mitchell CE, Agrawal AA, Bever JD, Gilbert GS, Hufbauer RA, Klironomos JN, Maron JL, Morris WF, Parker IM, Power AG, Seabloom EW, Torchin ME, Vazquez DP. 2006. Biotic interactions and plant invasions. Ecology Letters 9: 726–740.

Rejmanek M, Richardson DM. 1996. What Attributes Make Some Plant Species More Invasive? Ecology 77: 1655–1661.

Ricciardi A, Atkinson SK. 2004. Distinctiveness magnifies the impact of biological invaders in aquatic ecosystems. Ecology Letters 7: 781–784.

Richards CL, Bossdorf O, Muth NZ, Gurevitch J, Pigliucci M. 2006. Jack of all trades, master of some? On the role of phenotypic plasticity in plant invasions. Ecology Letters 9: 981–993.

Sher AA, Hyatt LA. 1999. The Disturbed Resource-Flux Invasion Matrix : a new framework for patterns of plant invasion. Biological Invasions 1: 107–114.

Simberloff D, Gibbons L. 2004. Now you See them, Now you don’t! – Population Crashes of Established Introduced Species. Biological Invasions 6: 161–172.

Simberloff D, Von Holle B. 1999. Positive Interactions of Nonindigenous Species: Invasional Meltdown? Biological Invasions 1: 21–32.

Stohlgren TJ, Jarnevich C, Chong GW, Evangelista PH. 2006. Scale and plant invasions: A theory of biotic acceptance. Preslia 78: 405–426.

Te Beest M, Le Roux JJ, Richardson DM, Brysting AK, Suda J, Kubesova M, Pysek P. 2012. The more the better? The role of polyploidy in facilitating plant invasions. Annals of Botany 109: 19–45.

Weiher E, Keddy PA. 1995. Assembly Rules, Null Models, and Trait Dispersion: New Questions from Old Patterns. Oikos 74: 159.

Williamson MH, Brown KC. 1986. The analysis and modelling of British invasions. Philosophical Transactions of the Royal Society of London. B, Biological Sciences 314: 505–522.
